# Mineral data (SEM, electron microprobe, Raman spectroscopy) from epithermal hydrothermal alteration of the Miocene Sigri Petrified Forest and host pyroclastic rocks, Western Lesbos, Greece

**DOI:** 10.1016/j.dib.2019.103987

**Published:** 2019-05-17

**Authors:** Georgia Pe-Piper, Alexis Imperial, David J.W. Piper, Nickolas C. Zouros, George Anastasakis

**Affiliations:** a Department of Geology, Saint Mary's University, Halifax, Nova Scotia B3H 3C3, Canada; b Geological Survey of Canada (Atlantic), Bedford Institute of Oceanography, PO Box 1006, Dartmouth, Nova Scotia B2Y 4A2, Canada; c Geography Department, University of the Aegean, Natural History Museum of the Lesvos Petrified Forest, 17 8th November Str., Mytilene 81100, Lesvos, Greece; d Section of Historical Geology-Palaeontology, Department of Geology & Geoenvironment, National and Kapodistrian University of Athens, Panepistimiopolis, Zografou 15784, Greece

**Keywords:** Opaline silica, Manganese oxides, Raman spectroscopy, Mineral composition, Tuff, Silicified wood

## Abstract

Data available from a detailed mineralogical investigation of the Petrified Forest of Lesbos and its host pyroclastic rocks [1] are summarized and a link is provided to the full data at https://data.mendeley.com/datasets/dxwfd32zms/1. Samples were taken from petrified wood, fresh and devitrified tuffs, and from epithermal veins and epithermally altered tuffs. Backscattered electron (BSE) images were made by scanning electron microscope (SEM) from polished thin sections of 16 samples to show textural relationships between minerals. Minerals were identified by energy dispersive spectroscopy (EDS). Further chemical analysis by electron microprobe (EMP) were made of trace elements in the petrified wood and of Mn-oxide minerals. Polymorphs of silica were investigated by Raman spectroscopy. SEM X-Ray maps were made of selected sites with manganese oxide minerals. In this contribution, the general character of each analyzed sample is summarized and a brief inventory of available data is presented, with specific reference to features in the on-line data. The significance of these data for the origin of the petrification of the wood and the epithermal veining of the host pyroclastic rocks is provided in “Nature of the hydrothermal alteration of the Miocene Sigri Petrified Forest and host pyroclastic rocks, Lesbos, Greece” [1] https://doi.org/10.1016/j.jvolgeores.2018.11.018. The data will be of comparative value to those investigating petrification of wood, devitrification of tuffs, and epithermal Mn-Fe mineralization in other areas.

**Table:** Specifications Table

Subject area	*Geology*
More specific subject area	*Hydrothermal minerals*
Type of data	*Tables and images*
How data was acquired	*SEM (TESCAN MIRA 3 LMU Variable Pressure Schottky Field Emission Scanning Microscope), EMP (JEOL-8200 electron microprobe) and Laser Raman Microspectroscopy (Horiba Jobin-Yvon LabRam HR confocal instrument)*
Data format	*SEM-EDS has raw analytical totals and individual element oxides recalculated to stoichiometric mineral totals without volatiles (so raw data can be back-calculated). Backscattered electron images are raw data with annotations of mineral types. EMP-WDS analyses have raw oxide data. Raw Raman spectroscopy intensity plots are presented (standard method for mineral analyses).*
Experimental factors	*Polished thin section (carbon coated)*
Experimental features	*Polished thin sections were analyzed by SEM using backscattered electron images to locate minerals and energy dispersive spectroscopy to determine their composition; composition of selected minerals was determined using wavelength dispersive spectroscopy on an EMP. Selected minerals were also analyzed by Laser Raman Microspectroscopy.*
Data source location	*Sigri Pyroclastic Formation, western Lesbos, Greece, details in Table 1*
Data accessibility	https://data.mendeley.com/datasets/dxwfd32zms/1
Related research article	*G. Pe-Piper, A. Imperial, D.J.W. Piper, N.C. Zouros, G. Anastasakis, Nature of the hydrothermal alteration of the Miocene Sigri Petrified Forest and host pyroclastic rocks, Lesbos, Greece. Journal of Volcanology and Geothermal Research 369 (2019), 172–187.*https://doi.org/10.1016/j.jvolgeores.2018.11.018*[1]*

**Table:** 

**Value of the data** • These data on detailed mineralogy of petrified wood samples, devitrified tuffs, and regional epithermal veins are useful for comparison with other areas of similar epithermal mineralogy. Data on the silica polymorphs can be compared with other volcanic successions and petrified wood. • The data will be of value to others working on epithermal veins and petrified wood, particularly with abundant manganese and iron (hydr)oxides. • Manganese and iron (hydr)oxides and devitrified volcanic glass have quite variable chemical compositions. The presentation of a few representative analyses in the summary paper [1] does not fully represent the range of data. Further investigations elsewhere may reveal new meaning to our data variability.

## 1 Data

Mineral data are presented for 16 strategically selected samples. Each sample is briefly summarized below, highlighting the mineralogical significance of the sample. Figure references refer to Appendices 1-4 in https://data.mendeley.com/datasets/dxwfd32zms/1. The content of these appendices is as follows: **Appendix 1**: Scanning Electron Microscope (SEM)-Back Scattered Electron (BSE) images and Energy Dispersive Spectroscopy (EDS) mineral analyses; **Appendix 2**: Raman Spectroscopy; **Appendix 3**: SEM X-ray maps; **Appendix 4**: Electron Microprobe (EMP) mineral analyses. For convenience, we refer to fine-grained mixtures of amorphous silica and iron (hydr)oxides as FeSi mixture, and of amorphous silica and manganese (hydr)oxides as MnSi mixture. The terms “Fe-oxide” and “Mn-oxide” are used for mixtures of various oxide and hydroxide minerals, both in thin section where mineral crystallites can be seen, and in the field.

### 1.1 800a: Yellow zoned pipe from a fault zone

The sample is from a mineralized pipe in a fault zone that shows three concentric zones (Fig. 1-1.1c). The outer zone is tuff with feldspar and quartz, the middle zone is FeSi ± MnSi mixtures, and the core of the sample consists of two types of silica: amorphous and microcrystalline (e.g. Fig. 1-1.3). Amorphous silica also forms veinlets (Fig. 1-1.2). The FeSi mixture is very fine grained in the middle zone (Fig. 1-1.1c), but more crystalline in the outer zone.

### 1.2 800b: brown fragments of vein from a fault zone

The sample consists of fragments of a complex vein in a fault zone cutting tuff (Fig. 1-2.1). It is predominantly a fine grained FeSi mixture cut by silica veinlets (Fig. 1-2.2, 2.6). Small grains of magnetite and feldspar are present.

### 1.3 806: Brown vein cutting lithic clast in tuff

The sample is a lithic clast in a vitric-lithic tuff cut by a brown vein (Fig. 1-3.1a). The host tuff includes K-feldspar, plagioclase (oligoclase-andesine), quartz, diopside, Ti-magnetite and rare ilmenite. Three types of veinlet are present: (I) MnSi mixture; (II) FeSi mixture with FeO_t_ < 60%; (III) FeSi mixture with FeO_t_ > 60%. The sequence of events was: Silica → brecciation of silica (Fig. 1-3.16) → FeSi mixture follows mineral pathways, e.g. crystal outlines, cleavage planes, fractures (Fig. 1-3.3) or direct deposition (veins II and III; Figs. 1-3.12, 17, 18) → MnSi mixture (vein I, Fig. 1-3.12). Original hornblende crystals are highly mineralized (Fig. 1-3.22) and diopside crystals are rimmed with MnSi mixture ± FeSi (Fig. 1-3.15a).

### 1.4 807: Yellow vein cutting tuff

This sample mostly consists of FeSi mixture cutting lithic tuff (Fig. 1-4.1a). MnSi mixture and silica veins (Fig. 1-4.2) are also present. The main silica vein is cross-cut by many silica veinlets (Fig. 1-4.19). The FeSi mixture shows botryoidal texture (Fig. 1-4.14) and more porous areas give a dark backscatter response (Fig. 1-4.24).

### 1.5 819: Yellow cement in vitric-lithic tuff

The sample is a vitric (pumice)-lithic tuff with a yellow color developed in some of the pumice fragments (Fig. 1-5.1b) that comprise a FeSi mixture with smectite (Figs. 1-5.4, 1-5.5, 1-5.6). Pyroclastic minerals, including those in lithic clasts, are: hornblende, oligoclase, andesine, labradorite, biotite, ilmenite, Ti-magnetite, and apatite. Secondary minerals include smectite, silica, hematite, and FeSi mixture. Hornblende has partly broken down to Ti-magnetite and andesine (Fig.1-5.9). The pumice fragments are crystal rich, with feldspars and green hornblende, and also contain small (∼100 μm), lithic clasts of volcanic rock (phenocrysts of hornblende and feldspars in a hyalopilitic groundmass) and a holocrystalline igneous rock (?enclave) made up mostly of hornblende and feldspar (Figs. 1-5.8).

### 1.6 839: White clay

The sample is a lithic-vitric-crystal tuff that has been altered to smectitic clay with some silica (Fig. 1-6.1a). Pyroclastic mineral crystals, mostly in lithic clasts, are K-feldspar, oligoclase, andesine, labradorite, apatite, zircon, ilmenite, rutile and quartz. Alteration minerals are smectite, silica, monazite, and a titania mineral. High-SiO_2_ smectite analyses result from mixture with amorphous silica (Figs. 1-6.7, 17, 20).

### 1.7 840a: Fresh ignimbrite

The sample is a fresh vitric-crystal welded ignimbrite (Fig. 1-7.1a). Pyroclastic crystals include biotite, sanidine, oligoclase, andesine, labradorite, zircon, apatite, ilmenite and Ti-magnetite. Alteration minerals include smectite, nontronite?, silica, and hematite (Figs. 1-7.7, 1-7.9, 1-7.17). Both feldspars (plagioclase, K-feldspar) and glass alter to smectite and silica. Euhedral laths (Fig. 1-7.26) or acicular crystals (Fig. 1-7.27) of barite postdate smectite or fill voids in glass (Fig. 1-7.16). Supplementary Table 4 of [1] gives chemical composition of glass, Supplementary Table 5 of [1] gives chemical composition of K-feldspar.

### 1.8 842: Black clay

The black clay sample (Fig. 1-8.1b) is predominantly smectite with small grains of silica (quartz), andesine and K-feldspar and lesser Ti-magnetite, ilmenite and biotite, of pyroclastic origin (Figs. 1-8.5, 1-8.8). Alteration minerals are smectite, silica, hematite and a titania mineral. Smectite is intermixed with abundant quartz crystallites (Figs. 1-8.9, 1-8.17).

### 1.9 843: Red clay

The red clay sample (Fig. 1-9.1a) is mostly smectitic clay with highly altered lithic clasts and crystals replaced by smectite + hematite (Figs. 1-9.6, 1-9.8, 1-9.21). Some quartz, mica, Fe-oxide/hydroxide and Ti-magnetite crystals are present.

### 1.10 844a: White highly altered ignimbrite

The bleached vitric-lithic-crystal ignimbrite (Fig. 1-10.1b) is the altered equivalent of fresh sample 840a. Pyroclastic minerals include K-feldspar, biotite (fresh and altered), zircon, rutile, ilmenite, and glass.

Devitrification has produced K-feldspar (adularia) and amorphous silica, in varying proportions in spherulites (Fig. 1-10.12, 17). In devitrification of glass shards, crystals nucleated from the margins of the shard (Figs. 1-10.3, 1-10.10). Alteration minerals include: kaolinite (Fig. 1-10.15), ilmenite (Figs. 1-10.6, 7), apatite (Fig. 1-10.7), adularia (Fig. 1-10.10), and silica (Figs. 1-10.8, 1-10.12).

Four textural types of biotite are present: (1) primary pyroclastic fresh-looking crystals (C) (Fig. 1-10.9); (2) Ragged crystals in euhedral blocky material (glass and K-feldspar) (CS) (Fig. 1-10.15); (3) Ragged crystals in altered ignimbrite (CAI) (Fig. 1-10.16); and (4) Crystals growing within vesicles (CV) (Fig. 1-10.7). Chemically, based on TiO_2_ (Supplementary Table 6 in Ref. [1]), three types of biotite crystals are present: (a) TiO_2_ ∼9% (CV); (b) TiO_2_ (5–6%) (C); and (c) TiO_2_ <1% (CS,CAI). Altered biotite crystals are partly silicified along their cleavages (Fig. 1-10.12).

Kaolinite has elevated contents of one or more of the elements SiO_2_, FeO, MgO, and K_2_O probably depending on what it is replacing. Both biotite and K-feldspar alter to kaolinite (Fig. 1-10.15). Zoned amygdules (Fig. 1-10.7) consist of an outer rim of kaolinite and tiny particles of ilmenite (6,7) and an inner core made up mostly of kaolinite (1). The amygdule show fractures and probable dissolution. Secondary apatite and probably ilmenite veinlets postdate the kaolinite.

### 1.11 844b: White highly altered ignimbrite

This sample (Fig. 1-11.1a) is similar to 844a. Devitrification involved development of spherulites (Figs. 1-11.4, 1-11.8, 1-11.22, 1-11.24) with various proportions of adularia and amorphous silica (Supplementary Table 7 in Ref. [1]). Devitrification of glass shards involved crystals nucleating from the margins of the shard (Fig 1-11.9). Hornblende crystals have been replaced by adularia and minor amorphous silica (Figs. 1-11.5, 1-11.31). A tentative relative age relationship of the alteration is: Glass alteration (devitrification, Fig. 1-11.9) → amorphous silica precipitation (Fig. 1-11.17), kaolinite (Figs 1-11.16, 1-11.27), and illite (Figs. 1-11.11, 1-11.12).

### 1.12 846a: White altered ignimbrite

The sample is a vitric-lithic-crystal ignimbrite that has been bleached and cut by numerous dark veins (Fig. 1-12.1a). Original pyroclastic minerals include biotite and sanidine. Three types of K-feldspar are identified chemically based on comparison with analyses from the fresh ignimbrite (840a) (Table 2 in Ref. [1]): I = Igneous crystals or crystals from igneous lithic clasts of sanidine; H = hydrothermal alteration to adularia from alteration of glass or feldspars; M = sanidine modified during the alteration. Glass devitrification formed adularia + amorphous silica (Fig. 1-12.11). Vein minerals are “Mn-oxide” and “Fe-oxide”. The “Mn-oxide” vein is zoned (Figs. 1-12.3, 4, 17) as detailed in Figure 6 of [1]. Kaolinite both predates (Figs. 1-12.6, 1-12.16) and postdates “Mn-oxide” (Fig. 1-12.21). K-feldspar crystals are partly replaced by kaolinite (Fig. 1-12.28).

### 1.13 846b: White altered ignimbrite

The sample is a bleached vitric-lithic-crystal ignimbrite with various mineralized veins (Fig. 1-13.1a). Jarosite and hematite fill open fractures in the rock, its lithic clasts and detrital crystals. Original pyroclastic minerals are oligoclase, andesine, K-feldspar, hornblende, ilmenite, and zircon. Alteration minerals are kaolinite, adularia, amorphous silica, jarosite, and hematite, the latter three in veins. Jarosite is a primary hydrothermal mineral with concentric crustiform growth (Fig. 1-13.5) and not an alteration product from pyrite (Fig. 1-13.3). Hornblende is replaced by silica and jarosite (Fig. 1-13.19), particularly along cleavage planes (Fig. 1-13.20). K-feldspar types are identified chemically as in sample 846a. Glass devitrification has produced amorphous silica and adularia (Fig. 1-13.21) and silica spherules (Figs. 1-13.6, 1-13.9, 1-13.33, 1-13.34). Feldspar crystals are replaced by kaolinite and silica (Fig. 1-13.22) or only kaolinite (Fig. 1-13.9). There is amorphous silica deposition around cavities (Fig. 1-13.8) or acicular crystals in the centre of hematite + jarosite spherules (Fig. 1-13.33, 1-13.34). In such spherules, the cores are made up of dark crystallites, then jarosite has precipitated and finally hematite next to a residual void (position b, Fig. 1-13.34). The same relationships have also been seen in cavities (e.g. Figs. 1-13.6, 1-13.8, 1-13.9). The relative age of alteration is: glass devitrification (Fig. 1-13.21) → amorphous silica precipitation (Figs. 1-13.8, 1-13.34) → jarosite (Fig.1-13.34) → hematite (Fig. 1-13.39). Kaolinite predates jarosite (Figs. 1-13.14, 1-13.17)

### 1.14 847: Yellow vein cutting ignimbrite

The sample is a yellow amorphous silica vein (Fig. 1-14.1b) with a few crystals of biotite and magnetite. A veinlet of FeSi mixture is present (Fig. 1-14.2). The main vein has a globular texture (Fig. 1-14.8). An inclusion of glass is altered to silica and smectite (Figs. 1-14.3, 4).

### 1.15 856-2 Petrified wood

The sample is petrified wood with layers of different colors (Fig. 1-15.1). Features are similar to 856-1. The observed colors are controlled by the amount of Fe present (red) and the relics of plant tissues (black) (Figs. 1-15.6, 28–32, 21).

### 1.16 856-1 Petrified wood

A different sample of petrified wood with different colors (Fig. 1-16.1). The color is influenced by the amount of Fe and organic matter present and is discussed in detail in Ref. [1] together with electron microprobe analyses of trace elements (Table 5 in Ref. [1]). Images of the various colors are: **Black** (Figs. 1-16.2-4; sites 1–3); **Greenish-yellow** (Figs. 1-16.5-6; sites 4–5); **Yellowish** (Figs. 1-16.7-10; sites 6–9); **Dark orange-red** (Figs. 1-16.11-15; sites 10–13); **Yellow-orange** (Figs. 1-16.16-17,21; sites 14,19); **White** (Figs. 1-16.18-20, sites 16,17,18).

## 2 Experimental design, materials, and methods

### 2.1 Field collection of samples

In the field, rock samples were collected from the Jithra ignimbrite, the sediments underlying the ignimbrite, hydrothermal yellow veins cutting across pyroclastic rocks or tree horizons, and small samples from fossilized trees from the Sigri Petrified Forest (Table 1). Samples were selected based on the presence of alteration during field observation, in order to identify hydrothermal mineral assemblages present in these rocks by comparison to unaltered rocks.

**Table 1:** Summary table of analytical techniques performed on investigated samples. Also available as Supplementary Data in Ref. [1].

Group	Sample number	Latitude	Longitude	Field rock name	XRD	SEM	EMP	LRM
Hydrothermal veins	800a	39.22895	25.89922	yellow nodule from a fault zone		X		X
	800b	39.22895	25.89922	brown fragment from a fault zone		X		
	806	39.22697	25.89908	hematite vein in pyroclastic rock	X	X		
	807	39.22590	25.89818	yellow vein in pyroclastic rock	X	X		
	847	39.22643	25.97270	yellow vein in ignimbrite	X	X		
	846a	39.24273	25.99203	"Mn-oxide" vein in white altered ignimbrite	X	X	X	X
	846b	39.24273	25.99203	"hematite" veins in white altered ignimbrite		X		
Fine-grained sediments	839	39.23975	25.99302	white clay	X	X		
	842	39.23975	25.99302	black clay	X	X		
	843	39.23975	25.99302	red clay	X	X		
Miscellaneous	815	39.22017	25.84165	silicified top of paleosol	X			
	819	39.22722	25.92183	yellow coloration in vitric-lithic tuff	X	X		
Jithra ignimbrite	840a	39.23975	25.99302	black glassy ignimbrite		X		
	844a/b	39.24273	25.99203	white altered ignimbrite	X	X		
Petrified wood	856-1	39.22958	25.90700	petrified wood		X		
	856-2	39.22958	25.90700	petrified wood		X	X	X

### 2.2 Sample Preparation

Sample preparation was mostly carried out in the Department of Geology at Saint Mary's University. All field samples were first visually examined to determine significant areas appropriate for cutting. The samples were cut using water and oil-cooled diamond blade rock saws and were sent to Vancouver Petrographics Ltd to be made into polished thin sections.

The polished thin sections of samples analyzed, using the scanning electron microscope (SEM) and the Electron Microprobe (EMP), were carbon coated using a Leica EM CED-030 carbon coater. In contrast, the carbon coating was cleaned off polished thin sections analyzed using the Laser Raman spectroscopy microscope (LRM), by gently rubbing methanol soaked Kim Wipes on the surface of the polished thin sections.

Samples analyzed for X-ray diffraction were prepared at Bedford Institute of Oceanography. The rock samples were crushed separately by placing them inside an iron crusher and by using a hammer to apply force until the materials were completely pulverized. Glass slides were cut into one inch squares and labeled on the underside. A smear slide was produced by spreading each sample mixed with methanol thinly and evenly across the one inch glass slide. The slides were air-dried until the methanol completely evaporated.

### 2.3 Analytical techniques

The samples were analyzed using a variety of techniques: (1) Scanning electron microscope (SEM) for chemical analyses, mineral identification and for textural relationships; (2) Electron microprobe (EMP) for quantitative chemical analyses of minerals; (3) Laser Raman microspectroscopy (LRM) for crystal structure determination; and (4) X-ray diffraction (XRD) analysis for mineral identification. The various analytical techniques used to analyze each sample is summarized in Table 1. Mineral abbreviations used are after Whitney and Evans [2].

#### 2.3.1 Electron microprobe (EMP)

A petrified wood sample and another sample containing “Mn-oxide” were analyzed at the Regional Electron Microprobe Centre at Dalhousie University, Halifax. Analysis was performed using a JEOL-8200 electron microprobe (EMP) which is equipped with a Noran 133 eV dispersive spectrometer and five wavelength spectrometers. This provides a more careful identification of the “Mn-oxide” minerals and a more accurate trace element identification of the petrified wood sample. Quartz and hematite standards were used for the wood; the same standards plus pyrolusite and chromite were used for the “Mn-oxide”. The equipment was operated at 15 kV.

#### 2.3.2 Scanning electron microscope (SEM)

SEM analysis was performed in the Regional Analytical Centre at Saint Mary's University using a TESCAN MIRA 3 LMU Variable Pressure Schottky Field Emission Scanning Microscope. The SEM is equipped with both a backscattered electron detector (BSE) and an INCA X-max 80 mm^2^ silicon drift detector (SDD) energy dispersive spectrometer (EDS) which are able to provide qualitative elemental/phase information and semi-quantitative elemental information respectively about the sample being analyzed. The EDS system has a detection limit of <0.1%. A pure cobalt plate was used as a standard to calibrate the beam for analysis. The beam has an average diameter of approximately <10 nm and has an X-ray production volume of ∼10 μm (depending on the minerals). The SEM has a maximum resolution of 1.2 nm at 30 kV. All analyses were acquired at 20 kV. Minerals were identified from EDS analyses using criteria summarized by Ref. [3] (their Table 2).

The Oxford Instruments INCA software is equipped with a QuantMap package which maps elements based on quantitative elemental compositional data. QuantMap data are presented as oxides.

#### 2.3.3 Laser Raman microspectroscopy (LRM)

Laser Raman spectroscopy analyses for samples containing SiO_2_ minerals were performed at the Regional Analytical Centre at Saint Mary's University using a Horiba Jobin-Yvon LabRam HR confocal instrument. The LRM uses a 100 mW 532 nm Nd-YAG diode laser from Toptica Phonotics and a Synapse charge-coupled device form Horiba Jobin-Yvon. The LRM uses a maximum of 100× Olympus MPlanN objective for image capture and analysis. The analyses were done in order to determine the silica polymorphs that are present. Raman spectra from Kingman and Henley [4] (their figure 1) and Ilieva et al. [5] (their figure 5) were used for comparison.

#### 2.3.4 X-ray diffraction analysis (XRD)

X-ray diffraction was carried out on random powder mounts prepared by crushing the sample and stirring it onto a glass slide with a little methanol, which was allowed to evaporate. Samples were run on a Siemens Kristaloflex diffractometer using Co Kα radiation. The air-dried samples were scanned from 2 to 52° 2θ, with a 0.2° step. Diffractograms were processed using EVA software. Minerals were identified from their characteristic d-spacing.

## References

[1] Pe-Piper G., Imperial A., Piper D.J.W., Zouros N.C., Anastasakis G. (2019). Nature of the hydrothermal alteration of the Miocene Sigri Petrified Forest and host pyroclastic rocks, western Lesbos, Greece. J. Volcanol. Geotherm. Res..

[2] Whitney D.L., Evans B.W. (2010). Abbreviations for names of rock-forming minerals. Am. Mineral..

[3] Pe-Piper G., Piper D.J.W., Wang Y., Zhang Y., Trottier C., Ge C., Yin Y. (2016). Quaternary evolution of the rivers of northeast Hainan Island, China: tracking the history of avulsion from mineralogy and geochemistry of river and delta sands. Sediment. Geol..

[4] Kingman K.J., Hemley R.J. (1994). Raman spectroscopic study of microcrystalline silica. Am. Mineral..

[5] Ilieva A., Mihailova B., Tsintsov Z., Petrov O. (2007). Structural state of microcrystalline opals: a Raman spectroscopic study. Am. Mineral..

