# Supplementary material for: Mineral data (SEM, electron microprobe, Raman spectroscopy) from epithermal hydrothermal alteration of the Miocene Sigri Petrified Forest and host pyroclastic rocks, Western Lesbos, Greece
Source: Data Brief. 2019 May 17;24:103987. doi: 10.1016/j.dib.2019.103987 (PMC6545405; doi:10.1016/j.dib.2019.103987)
Supplement: Multimedia component 1 [file mmc1.doc]

Conflict of Interest and Authorship Conformation Form

Please check the following as appropriate:

- STATEMENT IS CORRECT All authors have participated in (a) conception and design, or analysis and interpretation of the data; (b) drafting the article or revising it critically for important intellectual content; and (c) approval of the final version.
- STATEMENT IS CORRECT This manuscript has not been submitted to, nor is under review at, another journal or other publishing venue.
- STATEMENT IS CORRECT The authors have no affiliation with any organization with a direct or indirect financial interest in the subject matter discussed in the manuscript
- The following authors have affiliations with organizations with direct or indirect financial interest in the subject matter discussed in the manuscript:

NONE

Author’s name Affiliation
